# Parents’ attitudes and views regarding antibiotics in the management of respiratory tract infections in children: a qualitative study of the influence of an information booklet

**DOI:** 10.3399/bjgpopen18X101553

**Published:** 2018-05-02

**Authors:** Anne RJ Dekker, Esther de Groot, Tom Sebalj, Lucy Yardley, Jochen WL Cals, Theo JM Verheij, Alike W van der Velden

**Affiliations:** 1 GP Trainee and PhD Candidate, Julius Center for Health Sciences and Primary Care, University Medical Center Utrecht, Utrecht, Netherlands; 2 Postdoctoral Researcher, Julius Center for Health Sciences and Primary Care, University Medical Center Utrecht, Utrecht, Netherlands; 3 Psychology Student, Julius Center for Health Sciences and Primary Care, University Medical Center Utrecht, Utrecht, Netherlands; 4 Professor of Health Psychology, Academic Unit of Psychology, Faculty of Social and Human Sciences, University of Southampton, Southampton, UK; 5 GP and Postdoctoral Researcher, Department of Family Medicine, CAPHRI School for Public Health and Primary Care, Maastricht University, Maastricht, Netherlands; 6 Professor of Primary Care, Julius Center for Health Sciences and Primary Care, University Medical Center Utrecht, Utrecht, Netherlands; 7 Postdoctoral Researcher, Julius Center for Health Sciences and Primary Care, University Medical Center Utrecht, Utrecht, Netherlands

**Keywords:** General practice, anti-bacterial agents, child, respiratory tract infections

## Abstract

**Background:**

Respiratory tract infection (RTI) is the most common reason to consult a GP during childhood, and often results in unnecessary prescribing of antibiotics. Using an information booklet during the consultation has been shown to be a promising tool to reduce antibiotic prescribing. The influence of such information on parents’ views, knowledge, and expectations has not been investigated yet.

**Aim:**

To explore the reported attitude and knowledge of parents towards antibiotics and management of childhood RTI, as well as the added influence of an information booklet, as perceived by parents.

**Design & setting:**

Qualitative interviews were conducted with Dutch parents who consulted the GP with their child for RTI symptoms and received an information booklet.

**Method:**

Semi-structured interviews were audio-recorded, transcribed, coded, and analysed using framework analysis by open-axial coding and describing themes.

**Results:**

Eighteen parents were interviewed. Four themes were identified: prior reticence towards antibiotics; expectations of the consultation and trust in the GPs’ treatment decision; confirmation and reassurance by the booklet; self-management and future consultation intentions. Dutch parents felt reassured and more confident about their pre-existing reticent attitude towards antibiotic treatment; therefore, they thought their opinion and attitude had not really been changed by the booklet.

**Conclusion:**

In a low-prescribing country like the Netherlands, information should focus on enhancing self-efficacy and providing concrete safety-netting advice. For other countries with less reticence towards antibiotics, it is recommended that the knowledge, attitude, and perceptions of the population is studied, in order to be able to tailor interventions.

## How this fits in

Overprescription of antibiotics is common in children, and information booklets are expected to reduce prescribing. However, their influence on parents’ views and attitudes towards antibiotics is unknown. In this qualitative study, Dutch parents reported reticence towards antibiotic use when their children were diagnosed with RTI; they felt the booklet confirmed these pre-existing views, and made them more confident to 'wait and see'. Parents mainly expected reassurance from their GP, trusted their treatment decision, and appreciated the safety-netting advice in the booklet, but the concept of antimicrobial resistance seemed difficult to comprehend. Therefore, information supply should first be piloted against pre-existing views and knowledge in the population.

## Introduction

RTI is the most common reason why children consult a GP.^1^ RTIs are predominantly viral and self-limiting, therefore antibiotic treatment is often not recommended.^2–5^ However, overprescription of antibiotics has repeatedly been shown.^6^ Even in a low-prescribing country such as the Netherlands, about one third of antibiotic prescriptions for children with RTI are not in accordance with guidelines.^7,8^ Overprescription of antibiotics is a worldwide problem. Antibiotic consumption is directly related to bacterial resistance, unnecessary side effects, and medicalisation, all of which results in higher healthcare costs.^9–11^

Previous studies have shown that GPs’ antibiotic prescribing behaviour is influenced by several mechanisms, including uncertain feelings about the clinical outcome, problematic communication, feeling pressured by parents, and fearing that parents will not accept non-prescribing.^12舑15^ RTI consultations are challenging for GPs as they need to make a rational treatment decision, provide reassurance, and offer evidence-based information. Recent reviews showed that the use of patient information booklets during the consultation is a promising tool to reduce antibiotic prescribing and patients’ intention to re-consult.^16,17^ Use of an interactive booklet about childhood RTI was evaluated in the UK.^18^ A relevant issue is how such information influences parental perception, knowledge, and attitudes, which could also be dependent on contextual factors like the level of antibiotic use in a country.

A qualitative interview study was conducted with Dutch parents who visited the GP with their child with RTI and received an information booklet within the Rational Antibiotic use Kids (RAAK) trial.^19^ The reported attitude and knowledge of parents towards (antibiotic) management of childhood RTI was explored, as well as the influence and added value they perceived of the booklet.

## Method

### Setting

This qualitative study was performed as part of the RAAK cluster randomised controlled trial, the first Dutch trial aiming to reduce antibiotic prescribing for children with RTIs in primary care.^19^ The intervention consisted of a concise, internet-based training course for GPs and the provision of GPs with an information booklet for parents, without specific instructions how to use it during the consultation. The booklet contained the following information in simple text and pictograms: epidemiology of RTIs, their predominantly viral cause, the self-limiting prognosis, rationale to withhold antibiotics, and antibiotic-related problems, including bacterial resistance. Additionally, the booklet explained child-specific self-management strategies, and signs and symptoms indicating when to consult the GP. The information booklet is available from the authors on request.

### Participants

Parents of children included in the intervention arm of the trial were consecutively approached by telephone within 3 weeks of their index consultation, and were asked to take part in a telephone interview. All participants provided verbal informed consent. All interviews were transcribed verbatim and anonymised.

### Data collection

An interview guide was developed during an expert discussion. The questions were structured into five topics:

1. parents’ attitude and views on antibiotics before having read the information booklet, and what might have contributed to these;
2. parents’ general impression of the information booklet;
3. what parents learned from the information booklet, and what information they regarded as most useful;
4. parents’ perceived changes in attitude and/or views after having read the information booklet, and what was considered to have contributed to this change; and
5. how the information booklet might affect their expectations of antibiotics and their consultation intentions in the future.

Finally, parents were asked whether or not they received an antibiotic prescription for their child. Semi-structured interviews were conducted from March 2015 to May 2015. Data collection and analyses were conducted in parallel, and interviews continued until no new themes emerged. Interviews were carried out by a researcher with Skype (version 7.5); were audio-recorded with Pamela (version 4.9), or MP3 Skype recorder (version 4.11); and were transcribed verbatim.

### Analysis

The first four interviews were coded independently by three researchers, and discussed to minimise inconsistencies in coding and to adapt the interview guide when necessary. The coding scheme based on these interviews was discussed with a fourth researcher, and was adjusted several times until the final coding scheme was unanimously accepted. Framework analysis was used; open-axial coding was applied to relate codes to each other. Related codes were grouped and discussed with all researchers, and four themes were developed based on consensus.^20,21^ Different perspectives on the concepts in this study were sought by analysing the data with a group of researchers from different backgrounds: qualitative research, learning sciences, primary care research, and work in general practice.^22^ Nvivo software (version 10.0) was used for analysis.

## Results

Eighteen parents who received the booklet were interviewed, 16 mothers and two fathers, with a mean age of 34 years (range 29–38 years). The mean age of their child was three years (range 5 months–6 years). The mean number of siblings was 1.1 (range 0–4). Of these parents, two received an antibiotic prescription for their child during the consultation.

Overall, parents were very enthusiastic about the information booklet. They regarded the booklet as complete, attractive, concise, easy to read, and clear. Some stated they would absolutely keep the booklet as a reference, or share it with others:

'[…] *Later when I read everything at home I began to think, "Hey this is useful!" I even texted some information to a friend of mine. I quickly took a picture of one of the pages.'* (P06)

From the interviews four main themes emerged:

1. prior reticence towards antibiotics;
2. trust in the GP, and parental expectations of the consultation;
3. confirmation and reassurance by the booklet; and
4. self-management and future consultation intention.

These themes will be elaborated on in further detail.

### Prior reticence towards antibiotics

Almost all parents mentioned that they were reticent towards antibiotic treatment for their child before having read the information booklet. A few thought antibiotics were useful for all infections, to help their child recover more quickly. In general, parents believed antibiotics should be avoided for their child if possible:

*'I was not just going to administer antibiotics to my son without reason, only if it is really necessary. I was already sure of that, and with that attitude I went to see the GP.'* (P06)

Concomitantly, parents also showed a hesitation towards using any medicine, for example, paracetamol. They preferred to consider medication when the body itself seemed unable to fight the illness properly:

*'To be honest, I always had this view of using as little medication as possible or even avoiding them at all, unless it is necessary* […] *Well, I do believe in the self-healing abilities of the body. So I consider it as administering extra junk when it’s not necessary. If it really helps, I mean, if the body really needs it to get better, or at least get better faster, then it’s fine. But if it’s not really necessary, then I don’t want it.'* (P01)

Parents explained their reticence towards antibiotics with reference to antibiotic resistance. Most parents did seem to know something about resistance before having read the information booklet:

*'I know you should be cautious using antibiotics, and that is of course also because bacteria can form a resistance against it.'* (P02)

Half of the parents had understood resistance as a 'resistant human body', despite the information explaining development of 'resistant bacteria'. They mainly believed that using antibiotics can make the body resistant to any antibiotic. They thought that when they would really need antibiotics, treatment would have limited effect on them. These parents were often also unaware of the fact that antibiotics are ineffective against viral infection and, concomitantly, that most of their child’s RTIs are viral:

*'I always thought you yourself could become resistant to antibiotics.* […] *I mean the body itself, because the bacteria are in the body, right?* […] *So, if you use a lot of antibiotics you will slowly become resistant to them.'* (P03)

Sometimes parents considered the resistance of the body as a general problem with medicine, for example, with painkillers as well:

*'For something simple such as paracetamol, I do prefer not to take all that too much, because, I do not know, maybe your body gets used to it and then they do not have the effect that they could have.'* (P13)

A minority knew nothing about bacterial resistance, but also had a clear attitude towards antibiotic use:

*'I think they* [GPs] *prescribe it a little too quickly, maybe waiting to see what happens might be better* […] *I don’t really know why I think this way, I just believe that antibiotics are prescribed too quickly in general* […] *I didn’t know anything about resistance before I read the information booklet.'* (P12)

Most parents were unable to point to what their initial views and opinions were based on. They mentioned education, their GP, family and friends, and the media:

*'Well, it is mainly based on information from other mothers, who say, uh, well, that you shouldn’t use antibiotics on children, if for instance they only have a simple ear or respiratory tract infection, or an inflammation of the throat. This, because it will resolve naturally most of the time.'* (P16)

*'Well, what you read in the papers, people can become really ill with some kind of resistant hospital bacterium.'* (P18)

### Trust in GP and parental expectations of the consultation

Parents showed a high degree of trust in their GP; when the GP did not prescribe antibiotics, they concluded that this was not necessary. When antibiotics were prescribed then this was deemed necessary, or, at least, that it would be unwise not to use antibiotics in this particular situation:

*' *[…] *I have a really good relationship with my GP, and the GP knows how I think about antibiotics. The GP will only prescribe if it is really necessary, you know. In cases where it wouldn't work with just good care and no antibiotics.'* (P06)

Parents noticed that GPs nowadays are quite reticent to prescribe antibiotics:

*'I know that they* [GPs]* are not very keen on giving antibiotics to children.'* (P16)

Parents felt that their GP’s attitude towards antibiotics was in line with their own. Trust made them accept the GPs’ decision whether or not to prescribe antibiotics:

*'If the GP prescribes antibiotics, then I will trust him. I still might ask him for a second time whether it is really necessary, or whether we can wait and see for a few more days. Yes, I always ask just to be sure, and my GP always explains it clearly.'* (P12)

As a consequence, most parents did not expect an antibiotic prescription; however, they do expect reassurance and advice about symptoms from their GP when they are in doubt over how to manage the child’s illness:

*'I did not go to the GP to get antibiotics, I just wanted the GP to listen to his lungs, that kind of examination.'* (P04)

The booklet seemed to bring some understanding for those who did not fully understand the GP's choice to prescribe or not prescribe antibiotics. This was mainly due to the information about the ineffectiveness of antibiotics against viral infection and the disadvantages of antibiotic use:

*'I think that the booklet throws some light on it for many people: that it is not unwillingness of my GP, but that the antibiotic really is ineffective for this illness.'* (P07)

Most GPs provided the booklet at the end of the consultation and briefly mentioned the content; they advised reading it at home. Parents were satisfied with not discussing the booklet during the consultation because of its clear information:

*' *[…] *No, I can do without, the booklet was clear enough to read it on my own at home.' *(P18)

Only one parent wished the information booklet was explained more by the GP during the consultation; for this parent, the booklet contained a lot of new information.

### Confirmation and reassurance by the information booklet

When asked what new knowledge parents gained from the booklet, half of them said the information was not new but was useful to read. The information was a confirmation and better explanation of what they already knew or thought. It brushed up their knowledge, reinforced symptomatic management of their children, and provided confidence in the self-limiting character of RTIs:

*'Actually, it was mostly a confirmation of what I already knew. But it’s always good to read it again.'* (P01)

*'If someone asks me, "Why don’t you ask for antibiotics?" Yes, then I can say: "I don’t do that, because it is not the right solution" *[…] *I feel more confident, more convinced.' *(P15)

Some parents did learn new information from the booklet. They mostly indicated that they had learned that antibiotics do not work for all infections — only for bacterial and not for viral ones — and/or when to call the GP with alarming symptoms. Other new insights included the disadvantages of antibiotic use, such as resistance and side effects. To a lesser extent, information about symptom duration, fever, and self-management advice were considered new:

*'Yes, it provided some* [new information]; […] *all those viruses that go round now, for which it is useless to give antibiotics, and that is what I did not know.'* (P14)

Most of the parents did not feel their opinion about antibiotics or knowledge of RTIs had been changed by the booklet. They already were quite reticent towards antibiotics, and this view was confirmed by the information of the booklet. However, they felt they could better explain their opinion:

*'The booklet confirmed that I indeed have to be careful with antibiotics. But, that is what I already knew before. I just did not exactly know why. Now, I really understand why.'* (P06)

Despite the fact that parents often said that the booklet was easy to read and contained clear pictograms, it nevertheless appeared that they often misreported the information in the booklet. Viruses and bacteria were often mixed up in their explanation why antibiotics do not work; they, for example, said antibiotics only work for specific viruses. As earlier mentioned, the concept of bacterial resistance was often still not fully understood after reading the booklet:

*'Well the information that antibiotics have advantages and disadvantages was most useful to me *[…] *The advantage is that antibiotics, mmm, kill those viruses that make you ill, but, that those viruses can adapt themselves, which you actually need for your own resistance I think.'* (P06)

### Self-management and future consultation intention

The information about 'when to contact your GP' was regarded as most useful. Some parents thought that the booklet would change their consultation behaviour as a result. Some parents did not think the booklet would have that effect, as they were already reticent about consulting their GP:

*'Especially the last page, about when you should call the GP, was the most important for me. I mean, knowing when something is considered minor, and when it is actually necessary to call the GP.'* (P13)

*'Well, it did change something. I will probably not call the GP as quickly as before, because of the guidance when to call the GP from the booklet. Of course, I take these things into consideration, I mean, does my child have a fever, or not, is the fever persisting for three days now. I’ve always considered these things, but now I know that when my child starts coughing, I will wait a week and see how it turns out.'* (P16)

Although parents were quite reticent towards antibiotics and consulting the GP soon after the onset of symptoms, most parents do acknowledge that once symptoms persist, they start to worry. Such a consultation is not necessarily for antibiotics, but for reassurance that the illness is not severe, or for self-management options for symptom relief:

*'Well, the problem was that he wasn’t getting enough sleep and was also keeping us awake all night. So, consulting the GP is a way of seeking a solution, but it depends on the symptoms* […] *No, no, I didn’t consult to get antibiotics. I just wanted to know if it was just a common cold, or something with his ears, and whether there was something I could do about it.'* (P17)

## Discussion

### Summary

The information booklet confirmed parents’ pre-existing views towards antibiotics. Therefore, parents reported that it did not really change their attitude, but did make them feel more confident to 'wait and see' first. They valued that the booklet substantiated their prior reticence towards antibiotics, and provided a better understanding of the GPs’ treatment decision. Most parents trusted the GPs’ professionalism and valued their judgement about the severity and treatment of the illness, and they expected reassurance during the consultation. For some parents, the information that antibiotics are not effective for most RTIs and the concept of bacterial resistance were new, but they expected neither to change their attitude towards antibiotics. The antibiotic-related information, especially about bacterial resistance, seemed difficult to understand. Information about when to consult the GP was regarded as most relevant.

### Strengths and limitations

A previous qualitative study performed in the UK explored parents’ and clinicians’ views on the development, process evaluation, and implementation of an interactive information booklet for parents.^18,23^ The present study focused on gaining insight into parental reporting on how and why the information booklet influenced their views and attitudes. This information might be relevant to understand the intervention's effect, to know which elements were most relevant, and to further optimise the intervention. A limitation was that parents’ views and attitudes were not obtained before they had read the information booklet; after having read the booklet, these might have been difficult to recall and explain. A second limitation was that parents could interpret, favour, and recall information from the booklet in a way that confirmed their pre-existing beliefs. In addition, the parents visited GPs who had followed an online training programme in communication skills and prudent antibiotic prescribing for RTIs, which could have influenced the consultation and, thereby, the parents’ views. Finally, parents knew about the aim of the trial, and could have provided socially acceptable answers.

### Comparison with existing literature

It has been shown that GPs can interpret parental concerns as an implicit demand for antibiotics, which is thought to contribute to the overprescription of antibiotics.^7,14,24^ In the present study, parents reported their prior reticence towards antibiotics and high trust in the GP, which resulted in a limited influence of the information booklet on their views. This finding should be understood in the Dutch context, with its sober attitude concerning treatment (for example, the use of 'watchful waiting'), which might explain the low antibiotic use in comparison with other countries.^25^ Furthermore, the high continuity of care in Dutch primary care, where patients are registered at one practice and are often linked to one GP, is probably the basis for patients' trust in the GP’s non-prescribing decision. These elements are considered to be of high importance in promoting appropriate antibiotic use.^26^

This reticent attitude towards antibiotics could, apart from culture, also be caused by misunderstanding of the concept of 'resistance'. The idea that 'the less often children take medicine, the better they work' was described in an earlier study about the opinions of parents about analgesics for their children.^27^ Most parents were aware of antimicrobial resistance before having read the information booklet, but it appeared that most parents did not fully understood the concept, despite efforts to explain this clearly in the booklet. Brookes-Howell *et al* previously described that people in Europe are aware of the link between antibiotic use and resistance, with the misinterpretation of antibiotic resistance as a property of a 'resistant human body' rather than a property of bacteria. They suggested interventions emphasising the transferability of resistance, and the societal contribution individuals can make through more appropriate antibiotic use to limit bacterial resistance.^28 ^In the present study, it seemed difficult for parents to exactly understand what antimicrobial resistance entails, and therefore completely understanding the concept appeared not important in a prudent attitude towards antibiotics. This study showed that the general knowledge that antibiotics have disadvantages and side effects, and cause ‘resistance’, was enough to result in a reticent attitude.

The parents mentioned that the booklet was often not discussed during the consultation, but they did not regard this interactive use as necessary. In the study of Francis *et al*, the interactive use of their booklet also did not appear to be consistently implemented, but was regarded as important, contrary to the present study's findings.^18^ Not discussing the booklet during the consultation might save time, and allow the booklet to be distributed in the waiting room, or via the internet. However, in situations without continuity of care — for example out-of-hours care, where patients tend to ask for antibiotics more readily— it might be preferable to use the booklet interactively during the consultation.^16,26^

### Implications for research and practice

Educating parents specifically on the effectiveness of antibiotics and antimicrobial resistance seemed less relevant than trust in the GP, reassurance, and clear safety-netting. In countries with a comparable context of low antibiotic use, focus of information supply should be on enhancing self-efficacy and providing safety-netting advice. For other countries, the present authors recommend studying the population’s pre-existing knowledge, attitude, and perception towards antibiotic management for RTI, in order to tailor information supply. In countries, where a reticent attitude might be less ingrained, an information booklet could, for example, help patients understand their GP’s non-prescribing treatment decision.
